# Supplementary material for: Simultaneous Determination and Risk Assessment of Pyrrolizidine Alkaloids in Artemisia capillaris Thunb. by UPLC-MS/MS Together with Chemometrics
Source: Molecules. 2019 Mar 19;24(6):1077. doi: 10.3390/molecules24061077 (PMC6471392; doi:10.3390/molecules24061077)
Supplement: Supplementary file 1 [file molecules-24-01077-s001.pdf]

Supplementary Material

Simultaneous Determination and Risk Assessment of Pyrrolizidine Alkaloids in *Artemisia capillaris* Thunb. by UPLC-MS/MS Together with Chemometrics

Li-Hua Chen [1], Jun-Chi Wang [1], Qi-Lei Guo [2], Yue Qiao [1], Hui-Juan Wang [1], Yong-Hong Liao [1], Di-An Sun [1] and Jian-Yong Si [1,*]

[1] The Key Laboratory of Bioactive Substances and Resources Utilization of Chinese Herbal Medicine, Ministry of Education, Institute of Medicinal Plant Development, Chinese Academy of Medical Sciences & Peking Union Medical College, Beijing 100193, China; lihuachen0706@163.com (L.-H.C.); jcwang@implad.ac.cn (J.-C.W.); MOON100107qy@163.com (Y.Q.) ; whj200428@163.com (H.-J.W.); yhliao@implad.ac.cn (Y.-H.L.); dasun@implad.ac.cn (D.-A.S.)

[2] Agilent Technologies Co. Ltd. (China), No.3, Wang Jing Bei Road, Chao Yang District, Beijing 100102, China; guo-ql@163.com

* Correspondence: jysi@implad.ac.cn; Tel.: +86-010-5783-3299

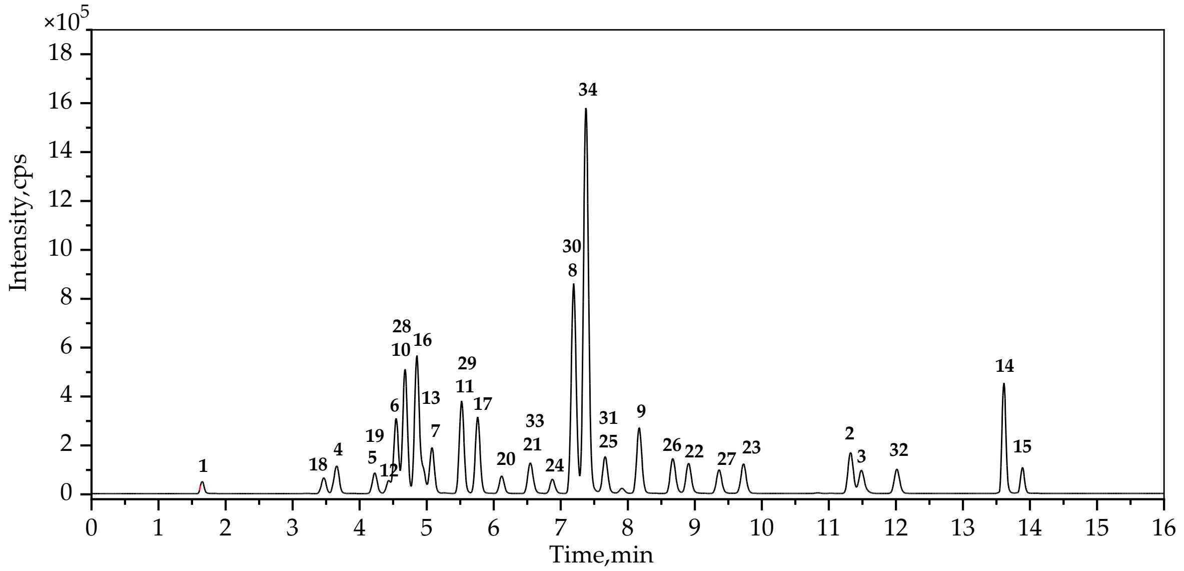

Figure S1. Total ion chromatogram with a mixed standard of PAs (50 μg/kg) by UPLC-MS/MS. **1:** Retronecine, **2:** Echimidine, **3:** Echimidine N-oxide, **4:** Erucifoline, **5:** Erucifoline N-oxide, **6:** Europine, **7:** Europine N-oxide, **8:** Heliotrine, **9:** Heliotrine N-oxide, **10:** Intermedine, **11:** Intermedine N-oxide, **12:** Jacobine, **13:** Jacobine N-oxide, **14:** Lasiocarpine, **15:** Lasiocarpine N-oxide, **16:** Lycopsamine, **17:** Lycopsamine N-oxide, **18:** Monocrotaline, **19:** Monocrotaline N-oxide, **20:** Retrorsine, **21:** Retrorsine N-oxide, **22:** Senecionine, **23:** Senecionine N-oxide, **24:** Seneciphylline, **25:** Seneciphylline N-oxide, **26:** Senecivernine, **27:** Senecivernine N-oxide, **28:** Indicine, **29:** Indicine N-oxide, **30:** 7-Acetylintermedine, **31:** 7-Acetylintermedine N-oxide, **32:** Senkirkine, **33:** Trichodesmine, **34:** 7-Acetyllycopsamine.

Retronecine-type:

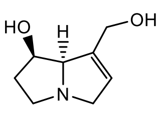

1

$C_8H_{13}NO_2$

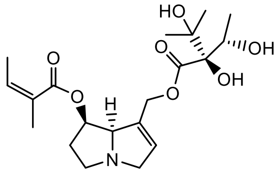

2

$C_{20}H_{31}NO_7$

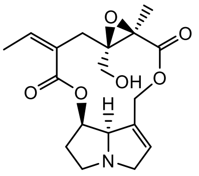

4

$C_{18}H_{23}NO_6$

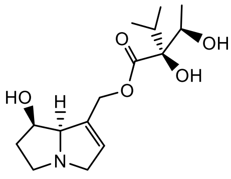

10

$C_{15}H_{25}NO_5$

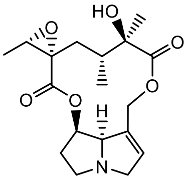

12

$C_{18}H_{25}NO_6$

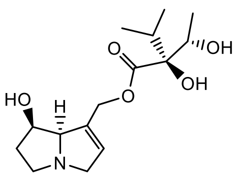

16

$C_{15}H_{25}NO_5$

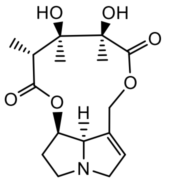

18

$C_{16}H_{23}NO_6$

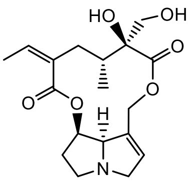

20

$C_{16}H_{25}NO_6$

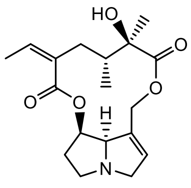

22

$C_{18}H_{25}NO_5$

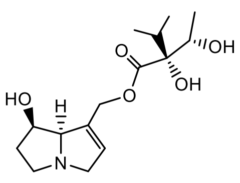

28

$C_{15}H_{25}NO_5$

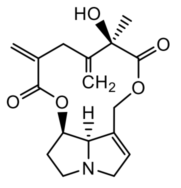

24

$C_{18}H_{23}NO_5$

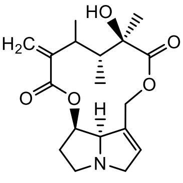

26

$C_{18}H_{25}NO_5$

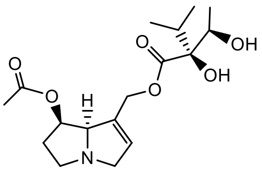

30

$C_{17}H_{27}NO_6$

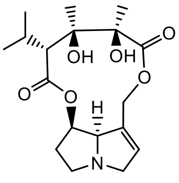

33

$C_{18}H_{27}NO_6$

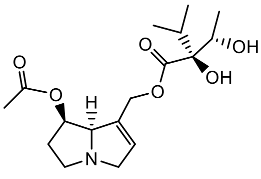

34

$C_{17}H_{27}NO_6$

Retronecine N-oxide-type:

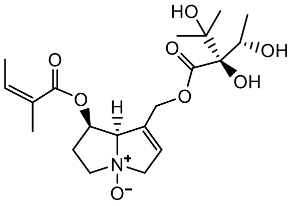

3

$C_{20}H_{31}NO_8$

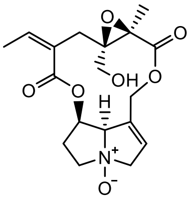

5

$C_{20}H_{31}NO_7$

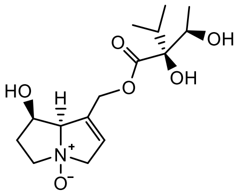

11

$C_{15}H_{25}NO_6$

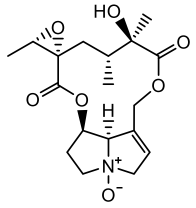

13

$C_{18}H_{25}NO_7$

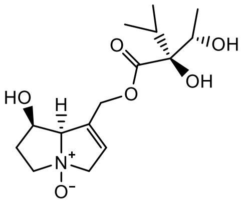

17

$C_{15}H_{25}NO_6$

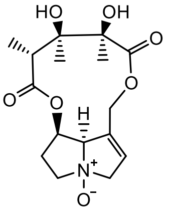

19

$C_{16}H_{23}NO_7$

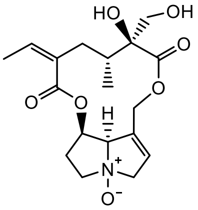

21

$C_{16}H_{25}NO_7$

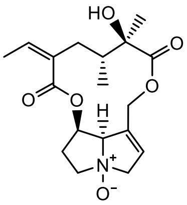

23

$C_{18}H_{25}NO_6$

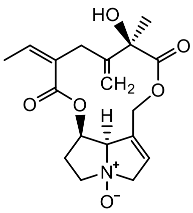

25

$C_{18}H_{23}NO_6$

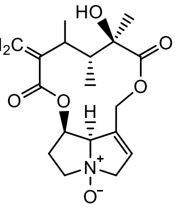

27

$C_{18}H_{25}NO_6$

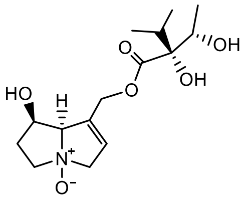

29

$C_{15}H_{25}NO_6$

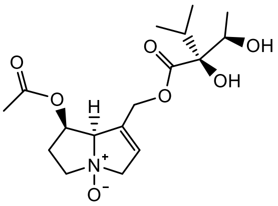

31

$C_{15}H_{25}NO_7$

Figure S2. *Cont.*

Heliotridine-type:

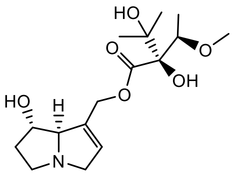

6
$C_{16}H_{27}NO_6$

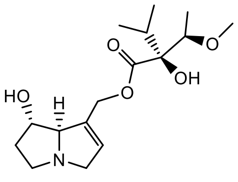

8
$C_{16}H_{27}NO_5$

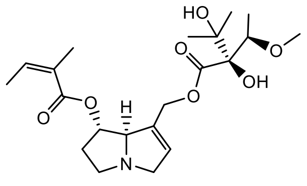

14
$C_{21}H_{33}NO_7$

Otonecine-type:

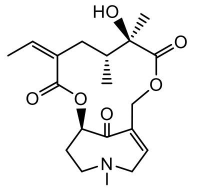

32
$C_{19}H_{27}NO_6$

Heliotridine N-oxide-type:

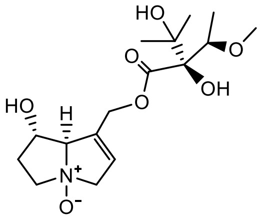

7
$C_{16}H_{27}NO_7$

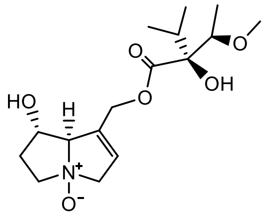

9
$C_{16}H_{27}NO_6$

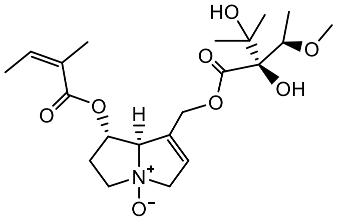

15
$C_{21}H_{33}NO_8$

Figure 2. Chemical structures of 34 PAs. **Retronecine-type:** **1**: Retronecine, **2**: Echimidine, **4**: Erucifoline, **10**: Intermedine, **12**: Jacobine, **16**: Lycopsamine, **18**: Monocrotaline, **20**: Retrorsine, **22**: Senecionine, **24**: Seneciphylline, **26**: Senecivernine, **28**: Indicine, **30**: 7-Acetylintermedine, **33**: Trichodesmine, **34**: 7-Acetyllycopsamine; **Retronecine N-oxide-type:** **3**: Echimidine N-oxide, **5**: Erucifoline N-oxide, **11**: Intermedine N-oxide, **13**: Jacobine N-oxide, **17**: Lycopsamine N-oxide, **19**: Monocrotaline N-oxide, **21**: Retrorsine N-oxide, **23**: Senecionine N-oxide, **25**: Seneciphylline N-oxide, **27**: Senecivernine N-oxide, **29**: Indicine N-oxide, **31**: 7-Acetylintermedine N-oxide; **Heliotridine-type:** **6**: Europine, **8**: Heliotrine, **14**: Lasiocarpine; **Heliotridine N-oxide-type:** **7**: Europine N-oxide, **9**: Heliotrine N-oxide, **15**: Lasiocarpine N-oxide; **Otonecine-type:** **32**: Senkirkine.

Table S1. The detailed recoveries of each PA with different SPE cartridges

No.	PCX [a]	C18 [b]	SCX [c]	C8/SCX [d]	X-C [e]	No.	PCX	C18	SCX	C8/SCX	X-C
1	75.38%	68.24%	107.46%	61.38%	76.89%	18	88.38%	77.05%	83.12%	88.79%	80.17%
2	79.41%	80.37%	84.99%	78.74%	92.89%	19	101.59%	54.40%	79.99%	64.66%	83.02%
3	91.38%	93.33%	73.38%	80.06%	89.81%	20	87.07%	76.64%	87.59%	71.32%	92.80%
4	71.82%	80.63%	58.14%	73.39%	69.63%	21	95.93%	92.52%	75.35%	86.44%	70.76%
5	87.15%	99.71%	48.29%	60.82%	79.91%	22	87.74%	77.44%	80.83%	93.37%	92.38%
6	97.25%	89.55%	91.41%	71.92%	64.50%	23	94.08%	94.42%	61.97%	75.08%	79.63%
7	95.74%	91.68%	88.87%	78.22%	84.72%	24	81.92%	78.45%	81.85%	87.88%	89.37%
8	92.67%	84.98%	91.15%	77.47%	86.62%	25	79.47%	88.94%	55.83%	73.25%	83.36%
9	93.88%	91.78%	85.49%	80.03%	89.48%	26	82.63%	76.95%	84.89%	88.32%	71.55%
10	91.45%	78.33%	93.52%	74.47%	77.45%	27	89.94%	89.14%	64.00%	81.20%	87.18%
11	96.50%	100.55%	82.27%	76.66%	99.71%	28	85.91%	77.58%	91.25%	74.47%	77.45%
12	80.18%	81.61%	83.69%	68.21%	66.15%	29	98.72%	98.81%	83.43%	76.66%	99.71%
13	80.67%	95.83%	63.02%	66.56%	83.66%	30	81.60%	80.03%	80.06%	75.49%	80.73%
14	89.46%	86.06%	88.41%	85.91%	80.41%	31	68.42%	109.95%	69.72%	71.87%	78.94%
15	97.82%	90.83%	87.93%	91.36%	77.02%	32	102.08%	86.41%	94.38%	82.21%	81.12%
16	85.91%	77.58%	91.25%	75.35%	76.72%	33	90.04%	74.12%	89.18%	60.20%	87.87%
17	98.72%	98.81%	83.43%	87.49%	91.73%	34	83.40%	80.27%	82.14%	71.16%	89.83%

a: Cleanert PCX b: Cleanert C18 c: Cleanert SCX d: Cleanert C8/SCX d: Strata X-C.

1: Retronecine, **2:** Echimidine, **3:** Echimidine N-oxide, **4:** Erucifoline, **5:** Erucifoline N-oxide, **6:** Europine, **7:** Europine N-oxide, **8:** Heliotrine, **9:** Heliotrine N-oxide, **10:** Intermedine, **11:** Intermedine N-oxide, **12:** Jacobine, **13:** Jacobine N-oxide, **14:** Lasiocarpine, **15:** Lasiocarpine N-oxide, **16:** Lycopsamine, **17:** Lycopsamine N-oxide, **18:** Monocrotaline, **19:** Monocrotaline N-oxide, **20:** Retrorsine, **21:** Retrorsine N-oxide, **22:** Senecionine, **23:** Senecionine N-oxide, **24:** Seneciphylline, **25:** Seneciphylline N-oxide, **26:** Senecivernine, **27:** Senecivernine N-oxide, **28:** Indicine, **29:** Indicine N-oxide, **30:** 7-Acetylintermedine, **31:** 7-Acetylintermedine N-oxide, **32:** Senkirkine, **33:** Trichodesmine, **34:** 7-Acetyllycopsamine.

Table S1-2. The concentrations of detected PAs with different extractant solvents (n = 3, ug/kg)

**Solvents**	**Methanol**	**0.05 M Acid-methanol**	**0.05 M Sulfuric acid**	**0.05 M Acid-ethanol**	**Ethanol**
Total PAs	133.1	104.0	88.8	85.4	57.6
	127.2	101.1	94.7	88.1	50.5
	140.0	110.2	92.6	90.0	65.4
Mean	133.4	105.1	92.0	87.8	57.8
SD	6.41	4.64	2.99	2.31	7.45

Table S2. MS/MS compound information and retention time (RT) of the PAs analytes

Num.	Compound Name	Abbr.	RT [b] (min)	Precursor Ion (m/z)	Product Ion (m/z)	Fragmentor (V)	CE [c] (V)
1	Retronecine	Ret	1.66	156.2	112 [a], 108.1	106	21
2	Echimidine	Em	11.41	398.2	220, 120 [a]	151	17
3	Echimidine N-oxide	EmNO	11.59	414.2	352.1, 254.1 [a]	152	33
4	Erucifoline	Er	3.66	350.2	138, 120 [a]	167	33, 29
5	Erucifoline N-oxide	ErNO	4.22	366.2	136 [a], 120.1	159	37
6	Europine	Eu	4.54	330.2	138 [a], 156	131	21, 33
7	Europine N-oxide	EuNO	5.07	346.2	172 [a], 111	144	33, 53
8	Heliotrine	He	7.19	314.2	156, 138 [a]	149	33, 21
9	Heliotrine N-oxide	HeNO	8.16	330.2	172, 138 [a]	149	29
10	Intermedine	Im	4.67	300.2	156, 138 [a]	144	33, 21
11	Intermedine N-oxide	ImNO	5.51	316.2	172 [a], 138	152	33
12	Jacobine	Jb	4.43	352.2	155, 120 [a]	167	33
13	Jacobine N-oxide	JbNO	4.94	368.2	296.1 [a], 120	157	25, 41
14	Lasiocarpine	Lc	13.61	412.2	336.2, 120 [a]	134	17, 33
15	Lasiocarpine N-oxide	LcNO	13.89	428.2	254.1 [a], 136.1	162	33
16	Lycopsamine	Ly	4.85	300.2	156, 138 [a]	149	33, 21
17	Lycopsamine N-oxide	LyNO	5.75	316.2	172 [a], 138	149	29
18	Monocrotaline	Mc	3.46	326.2	237.2, 120 [a]	157	30, 37
19	Monocrotaline N-oxide	McNO	4.21	342.2	137 [a], 118.1	167	33, 37
20	Retrorsine	Re	6.10	352.2	138, 120.1 [a]	177	33
21	Retrorsine N-oxide	ReNO	6.56	368.2	136, 118.1 [a]	172	41, 33
22	Senecionine	Sn	8.89	336.2	120.1 [a], 138	185	33
23	Senecionine N-oxide	SnNO	9.71	352.2	136, 118 [a]	167	37, 45
24	Seneciphylline	Sp	6.86	334.2	138, 120 [a]	162	29
25	Seneciphylline N-oxide	SpNO	7.64	350.2	136, 120.1 [a]	154	37
26	Senecivernine	Sv	8.66	336.2	138.1, 120 [a]	177	33
27	Senecivernine N-oxide	SvNO	9.35	352.2	136, 118.1 [a]	162	37
28	Indicine	Ic	4.67	300.2	156.1, 138 [a]	141	33, 21
29	Indicine N-oxide	IcNO	5.51	316.2	172 [a], 138	154	29
30	7-Acetylintermedine	7-Im	7.18	342.2	180, 120 [a]	144	17, 29
31	7-Acetylintermedine N-oxide	7-ImNO	7.65	358.2	214 [a], 137	157	29, 33
32	Senkirkine	Sk	12.13	366.2	168 [a], 150	157	33, 29
33	Trichodesmine	Td	6.52	354.2	222.1 [a], 120.1	162	33, 45
34	7-Acetyllycopsamine	7-Ly	7.36	342.2	180, 120.1 [a]	146	17, 25

a: Ions for quantitative determination; b: RT = retention time; c: CE = collision energy

Table S3. The concentration of each individual PA and the total contents in *A. capillaris* (mean ± SD, n=3, ug/kg)

ID	Im	Ly	ImNO	LyNO	Sk	EmNO	Sp	SpNO	Total PAs
Y1	3.61 ± 0.36	1.50 ± 0.18	9.36 ± 1.14	10.00 ± 1.95	0.75 ± 0.13	-	-	-	25.22 ± 3.77
Y2	3.35 ± 0.33	5.02 ± 0.40	24.81 ± 1.73	39.55 ± 2.45	-	-	-	-	72.73 ± 4.92
Y3	4.37 ± 0.35	6.37 ± 1.16	41.73 ± 2.52	89.35 ± 4.34	15.81 ± 1.60	-	-	-	157.63 ± 9.97
Y4	14.80 ± 1.70	36.21 ± 2.38	187.28 ± 7.33	580.69 ± 9.48	-	-	-	-	818.99 ± 20.90
Y5	6.85 ± 0.82	7.63 ± 0.31	28.86 ± 2.84	55.96 ± 4.34	-	-	-	-	99.31 ± 8.31
Y6	1.37 ± 0.26	-	2.94 ± 0.36	1.00 ± 0.09	-	-	-	-	5.30 ± 0.71
Y7	6.07 ± 0.54	3.17 ± 0.34	41.17 ± 4.30	27.46 ± 1.43	0.11 ± 0.02	3.25 ± 0.46	-	-	81.23 ± 7.09
Y8	-	7.34 ± 0.35	-	82.02 ± 2.70	0.10 ± 0.02	-	-	-	89.47 ± 3.07
Y9	15.48 ± 1.39	-	3.20 ± 0.18	1.39 ± 0.10	0.25 ± 0.05	-	1.35 ± 0.16	5.57 ± 0.35	27.24 ± 2.24
Y10	0.18 ± 0.03	-	0.60 ± 0.04	0.90 ± 0.07	0.15 ± 0.02	-	-	-	1.84 ± 0.16
Y11	0.22 ± 0.02	-	0.19 ± 0.03	0.56 ± 0.11	-	-	-	-	0.97 ± 0.16
Y12	0.71 ± 0.09	0.29 ± 0.03	2.28 ± 0.22	0.96 ± 0.13	0.18 ± 0.03	-	-	-	4.42 ± 0.50
Y13	0.56 ± 0.03	0.29 ± 0.05	4.38 ± 0.19	4.05 ± 0.14	0.32 ± 0.03	-	-	-	9.60 ± 0.44
Y14	0.11 ± 0.02	-	0.42 ± 0.04	0.13 ± 0.02	0.19 ± 0.01	-	5.14 ± 0.73	-	5.99 ± 0.82
Y15	0.20 ± 0.01	0.44 ± 0.04	0.79 ± 0.11	2.98 ± 0.36	0.20 ± 0.01	-	-	-	4.60 ± 0.53
Y16	-	-	-	0.11 ± 0.01	0.28 ± 0.01	-	-	-	0.39 ± 0.02
Y17	4.90 ± 0.27	3.17 ± 0.30	7.23 ± 0.46	6.32 ± 0.28	-	-	-	-	21.61 ± 1.29
Y18	0.16 ± 0.02	-	0.31 ± 0.02	0.79 ± 0.02	0.16 ± 0.03	-	-	-	1.41 ± 0.10
Y19	-	0.33 ± 0.03	-	0.11 ± 0.02	0.23 ± 0.04	-	-	-	0.67 ± 0.08
Y20	-	-	-	-	-	-	-	-	-
Y21	-	3.17 ± 0.45	-	0.95 ± 0.13	0.16 ± 0.03	-	-	-	4.28 ± 0.61
Y22	12.30 ± 0.62	92.05 ± 1.93	255.46 ± 5.56	1750.99 ± 14.46	0.41 ± 0.08	-	-	-	2111.22 ± 22.65
Y23	11.55 ± 1.57	1.94 ± 0.19	31.13 ± 2.68	7.58 ± 0.56	0.56 ± 0.06	-	-	-	52.76 ± 5.06
Y24	8.05 ± 0.78	14.15 ± 0.29	62.47 ± 1.91	135.65 ± 4.36	0.20 ± 0.04	-	-	-	220.51 ± 7.37
Y25	2.16 ± 0.15	0.52 ± 0.09	2.23 ± 0.20	1.33 ± 0.21	-	-	-	-	6.24 ± 0.65
Y26	1.10 ± 0.22	1.43 ± 0.19	14.64 ± 1.75	35.54 ± 0.61	0.29 ± 0.04	-	-	-	53.00 ± 2.81
Y27	3.32 ± 0.31	-	24.03 ± 1.51	1.62 ± 0.13	0.23 ± 0.02	-	-	-	29.20 ± 1.97
Y28	1.70 ± 0.10	0.73 ± 0.04	26.78 ± 1.68	3.04 ± 0.50	0.16 ± 0.02	-	-	-	32.41 ± 2.35
Y29	383.28 ± 5.05	35.01 ± 1.51	49.64 ± 3.57	16.30 ± 1.55	0.56 ± 0.06	-	-	-	484.80 ± 11.74
Y30	8.65 ± 1.06	19.86 ± 1.09	48.03 ± 1.48	353.38 ± 8.15	1.08 ± 0.11	-	-	-	430.99 ± 11.88

-: not detected.

Table S4. Detailed sources of thirty batches of *A. capillaris* samples

Batch Number	Source	Batch Number	Source
Y1	Beijing city	Y16	Linfen City, Shanxi Province
Y2	Datong City, Shanxi Province	Y17	Liupanshan, Ningxia Province
Y3	Baoding City, Heibei Province	Y18	Meizhou City, Guangdong Province
Y4	Shaoxing City, Zhengjiang Province	Y19	Guoluoxing City, Qinghai Province
Y5	Changsha City, Hunan Province	Y20	Zhengzhou City, Henan Province
Y6	Wuhan City, Hubei Province	Y21	Suqian City, Jiangsu Province
Y7	Anguo City, Heibei Province	Y22	Tianshui City, Gansu Province
Y8	Baoji City, Shaanxi Province	Y23	Huanggang City, Hubei Province
Y9	Bijie City, Guizhou Province	Y24	Hangzhou City, Zhengjiang
Y10	Bozhou City, Anhui Province	Y25	Shijiazhuang City, Hubei Province
Y11	Changzhi City, Shanxi Province	Y26	Hangzhou City, Zhengjiang
Y12	Hezhou city, Guangxi Province	Y27	Yishui City, Shandong Province
Y13	Linyi City, Shandong Province	Y28	Hangzhou City, Zhengjiang Province
Y14	Shigatse, Tibet	Y29	Ganzhou City, Jiangxi Province
Y15	Kunming City, Yunan Province	Y30	Longyan City, Fujian Province

Table S5. UPLC system configuration and parameters

**Configuration**	**Parameters**		
Pump	Two Agilent 1290 Infinity II binary pumps (p/n G7120A)		
Multisampler/autosampler	Agilent 1290 Infinity II multisampler 108-vial well-plate trays (p/n G7167B)		
Column compartment	Agilent 1290 Infinity thermostatted column compartment with a 2-position 10 port valve (p/n G7116B)		
Analytical column	Agilent EclipsePlus C18, 3.0 mm × 150 mm, 1.8 μm ZORBAX LC column (p/n 959759-302)		
Column temperature	40°C		
Needle Wash Mode	Flush Port		
Injection volume	2.00 μL		
Mobile phase A	0.05% Formic acid and 2.5 mM/L Ammonium formate water		
Mobile phase B	0.05% Formic acid and 2.5 mM/L Ammonium formate in methanol		
Flow rate	0.400 mL/min		
Gradient	Time (min)	A（%）	B（%）
	0.50	95	5
	1.00	80	20
	11.00	63	37
	13.00	5	95
	15.50	5	95
	15.60	95	5
Stop time	19.00 min		

Table S6. UPLC triple quadrupole mass spectrometer configuration and parameters

**Configuration**	
Instrument	Agilent 6470 triple quadrupole mass spectrometer with Agilent Jet Stream
Ion mode	ESI + Agilent Jet Stream
Scan Type	MRM
Delta EMV	300 V
Ionization mode	Positive
Dwell time	5 ms
Cell accelerator voltage	3 V
**Source Parameters**	
Drying gas temperature	300°C
Drying gas flow	7 L/min
Nebulizer pressure	40 psi
Sheath gas Heater	325°C
Sheath gas flow	11 L/min
Capillary voltage	3500 V
Q1/Q2 resolution	Unit/Enh
